# Are there any potentially dangerous pharmacological effects of combining ADHD medication with alcohol and drugs of abuse? A systematic review of the literature

**DOI:** 10.1186/s12888-015-0657-9

**Published:** 2015-10-30

**Authors:** Xanthe M. Barkla, Paul A. McArdle, Dorothy Newbury-Birch

**Affiliations:** Children and Young People’s Service, Villa 9, Northgate Hospital, Morpeth, Northumberland UK; Institute of Health & Society, Newcastle University, Newcastle, UK; School of Health & Social Care, University of Teesside Middlesbrough, Middlesbrough, UK

**Keywords:** Attention deficit hyperactivity disorder, Systematic review, Alcohol, Illicit drugs, Medication

## Abstract

**Background:**

Among young people up to 18 years of age, approximately 5 % have attention deficit hyperactivity disorder (ADHD), many of whom have symptoms persisting into adulthood. ADHD is associated with increased risk of co-morbid psychiatric disorders, including substance misuse. Many will be prescribed medication, namely methylphenidate, atomoxetine, dexamphetamine and lisdexamfetamine. If so, it is important to know if interactions exist and if they are potentially toxic.

**Methods:**

Three databases (Medline, EMBASE and PsychINFO) from a 22 year period (1992 – June 2014) were searched systematically. Key search terms included alcohol, substance related disorders, methylphenidate, atomoxetine, dexamphetamine, lisdexamfetamine, and death, which identified 493 citations (344 after removal of duplicates). The eligibility of each study was assessed jointly by two investigators, leaving 20 relevant articles.

**Results:**

We identified only a minimal increase in side-effects when ADHD medication (therapeutic doses) was taken with alcohol. None of the reviewed studies showed severe sequelae among those who had overdosed on ADHD medication and other coingestants, including alcohol.

**Conclusions:**

The numbers across all the papers studied remain too low to exclude uncommon effects. Also, studies of combined effects with novel psychoactive substances have not yet appeared in the literature. Nevertheless, no serious sequelae were identified from combining ADHD medication with alcohol/illicit substances from the pre-novel psychoactive substance era.

## Background

Among young people up to 18 years of age, approximately 5 % world-wide are said to have attention deficit hyperactivity disorder (ADHD), a neurodevelopmental syndrome of impulsiveness, inattention, and overactivity that can result in long-term educational and social disadvantage [1, 2]. Symptoms persist into adulthood for a significant minority [3]. Co-morbid substance use disorders (SUD) involving alcohol and cannabis misuse but also stimulants, depressants, hallucinogens and now novel psychoactive substances (NPS) or ‘legal highs’ emerge in adolescence [4]. Young people with ADHD are significantly more likely to develop SUDs than those without ADHD [5].

Many people with ADHD are prescribed medication for their symptoms, particularly stimulants, (often methylphenidate and dexamphetamine) and atomoxetine (NICE 2006). If an individual is suspected of misusing substances, it is crucial to understand if it safe also to prescribe medication. We contacted the manufacturers who recommended that stimulants and atomoxetine not be coingested with alcohol or illicit substances. They had no specific information on potential interactions.

There is limited empirical evidence to guide whether it is advised to treat ADHD before, simultaneously, or only after remission of SUDs [6, 7]. Current guidelines suggest that cannabis use may not be a contraindication to pharmacological treatment but due to shared neurochemical mechanisms, and while not explicitly mentioned, certain novel psychoactive substances (NPAs), ‘cocaine is likely to be a real hazard’ [8]. In the absence of such information, faced with adolescents misusing substances the tendency is for clinicians to avoid prescribing. As adolescents with untreated compared to treated ADHD may have poorer long-term outcomes, this may leave the most vulnerable without adequate intervention [9].

Methylphenidate and dexamphetamine are sympathomimetic agents thought to act both in the central nervous system (CNS) and peripherally by enhancing dopaminergic and noradrenergic transmission through blockade of relevant transporters [10]. There is theoretical potential and some animal evidence for additive effects between prescribed and non-prescribed sympathomimetic agents [11, 12] potentially resulting in a toxic sympathomimetic syndrome with prominent cardiac and neurological effects [10, 12]. Such a syndrome may underly so-called ‘excited delirium’; agitation, paranoia, hyperthermia and muscle breakdown, symptoms similar to neuroleptic malignant syndrome [13]. However, there is also evidence that different stimulants may compete for receptor binding sites limiting the potential for additive effects [14].

Atomoxetine is a selective noradrenaline reuptake inhibitor metabolised by the hepatic oxidase system [15]. It has a chemical structure that resembles the SSRI fluoxetine, that has been implicated in the emergence of mania or the serotonin syndrome, comprised of mental, autonomic and neurological effects [16]. This suggests that toxic or tolerability effects could arise in alcohol misusing individuals [17], through additive effects with stimulants or with NPS that impact serotonin receptors.

## Methods

We systematically searched three databases (Medline, EMBASE and PsychINFO) from the past 22-years (1992 – June 2014) using PRISMA guidelines [18]. Only those articles that had abstracts available in the English language were included. We used the key search terms alcohol, substance related disorders, death, and combined these with methylphenidate, atomoxetine, dexamphetamine and lisdexamfetamine in turn.

Titles and abstracts of all articles found were screened by XB and PM. Full text articles were screened by XB and PM. The references of all full text articles included were also hand searched to identify any other potentially relevant articles. Study quality was assessed using the relevant tools from Critical Appraisal Skills Program (CASP) or the STROBE document as appropriate to the study type [19].

We included articles which mentioned the coingestion of ADHD medication (methylphenidate, dexamphetamine, lisdexamfetamine and atomoxetine) with another substance including alcohol, publications relating to animal models, and, due to the paucity of data regarding adolescents, all age groups. Also, to clarify mechanisms that might underlie toxicity, we included certain articles covering the physiological effects of ADHD medication alone. We excluded articles relating to the abuse of ADHD medication alone, case reports, articles relating to exposure of illicit drugs or alcohol in utero, foetal alcohol syndrome, and to cigarette smokers only.

Our main outcome was to identify any potential effects (whether positive or negative) on an individual should they combine their ADHD medication with alcohol or an illicit substance. We acknowledge the dangers of diversion of stimulant medication in the substance misuse population; however our study did not focus on this element.

## Results

**Fig. 1:**
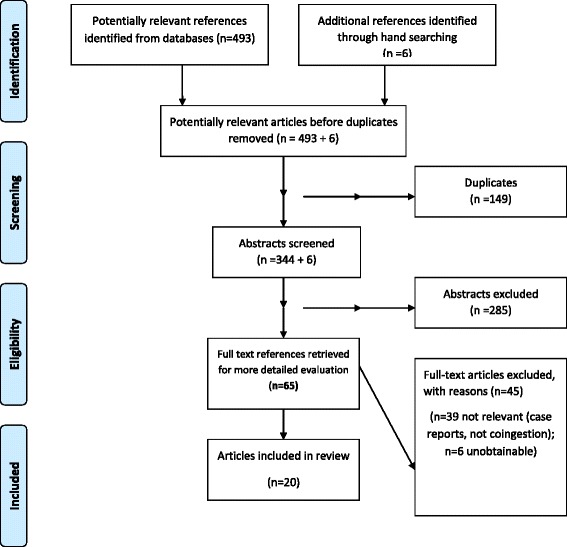
Flow chart of systematic review and study selection

The search strategy identified 493 citations (344 after removal of duplicates), of which 59 were potentially relevant. We also obtained 6 other articles from hand searching the references, providing 65 articles (Fig. 1). Twenty articles met inclusion criteria. In relation to bias, six of the included studies were found to have a low risk of bias [20–25], four a high risk [26–29] and 10 an unsure risk of bias [17, 30–38].

## Discussion

### Methylphenidate and alcohol

There was one animal study of methylphenidate and alcohol [31] and four of the combination in humans [28, 29, 34, 36]. The animal study reported that methylphenidate reduced the consumption of ethanol by mice and when simultaneously injected, ethanol and methylphenidate interact to increase locomotor stimulation and ataxia [31].

One human study was laboratory based and suggested that when delivered experimentally by mouth, the combination was well tolerated without serious adverse effects [34]. Two studies of users reported that alcohol ingestion often preceded that of methylphenidate and, interspersed with methylphenidate, continued over repeated administrations [28, 29]. Participants reported consuming significantly more alcohol when used in conjunction with methylphenidate relative to when they used alcohol alone. Combined methylphenidate-alcohol use was described as producing euphoria, energy and a diminished sense of drunkenness. Some likened the experience to using alcohol with cocaine. A final human study combined data from randomized controlled trials of methylphenidate treatment of ADHD among substance users. This reported that adolescents with higher baseline use of ethanol were more likely to have gastrointestinal symptoms (not further defined) relative to placebo [36]. However there was no significant difference in serious adverse events between treatment groups. They found no increase in the diversion of methylphenidate in the substance misuse versus the placebo group.

### Methylphenidate with other drugs

Two trials of methylphenidate among users of other amphetamine and cocaine [35, 39] and one experimental toxicology study reported no serious adverse effects [36]. However, cocaine users taking methylphenidate reported loss of the positive subjective effects of cocaine and more sadness, euphoria and insomnia. A meta-regression [25] and an RCT [40] reported that as a treatment for ADHD, methylphenidate was ineffective in the presence of SUD.

Two accident and emergency toxicology studies reported no additional toxicity when methylphenidate was combined with other agents [27, 32]. A randomized cross-over placebo controlled trial reported that, in the main, the combination of methylphenidate produced MDMA type effects but increased blood pressure to a greater degree than either alone [14].

### Dexamfetamine

Two studies examined combinations of dexamfetamine with alcohol [22, 38]. As with methylphenidate, participants reported that using dexamphetamine made it possible to ‘drink like a trooper’ while socializing for longer, with less perceived drunkenness or loss of control than with alcohol alone.

### Atomoxetine

Two studies explored combinations of atomoxetine and alcohol [17, 21]. Nausea was more likely in heavy drinkers taking atomoxetine, however there were no other differences in treatment emergent side effects.

## Conclusions

As diagnoses of ADHD have increased among young people of substance using age, the problem of prescribing for substance users with ADHD has become a common dilemma [31]. This review explores current available information concerning risks of toxicity attached to pharmacological treatment of ADHD in the context of substance misuse.

On the basis of experimental studies, and studies of alcohol dependent individuals, when methylphenidate, dexamphetamine or atomoxetine are ingested orally with alcohol, acute severe side-effects appear uncommon. However, as co-ingestion of stimulants such as methylphenidate or amphetamine with alcohol may enable more ‘late night partying’ and high levels of alcohol consumption, there is enhanced risk of more chronic harm from alcohol toxic effects [29, 31]. We did not identify serious adverse side-effects in those with poly-drug misuse using ADHD medication, even in overdose.

Despite the theoretical possibility of serotonergic, hyperdopaminergic or other hyper stimulated states, on the basis of limited evidence, apart from one study suggesting hypertension following a combination of methylphenidate and MDMA, ADHD medication does not normally add to toxicity of alcohol or illicit substances from the pre-novel psychoactive substance (NPS) era.

Limitations include the relatively small numbers in the studies cited. In part, this may reflect a fundamental problem with the science highlighted by Bauman [41]; banning psychoactive agents seriously impedes research into the effects of substances in widespread use alone and in combination by large numbers of young people. In addition, there is no data concerning combinations of cannabis and ADHD medications. However, as cannabis is ubiquitous in the substance using community, it is likely that many adolescents prescribed medications are also using cannabis apparently without acute mishap. However, we lack data concerning any longer term problems with this practice. The effect of combining ADHD medication and NPS is unknown and the numbers in published studies are too few to exclude unusual reactions. ADHD medication appears less effective or even ineffective among those with SUD and if nevertheless prescribed, its use, efficacy and combination with other substances should be closely monitored in an appropriately cautioned consumer.

With appropriate caution, including explanations of the limits of the science, it appears that a blanket contraindication is not justified when considering treatment of ADHD in selected substance users.
